# Comprehensive analysis of blood proteome response to necrotic enteritis in broiler chicken

**DOI:** 10.1186/s13567-025-01519-7

**Published:** 2025-04-24

**Authors:** Svitlana Tretiak, Teresa Mendes Maia, Tom Rijsselaere, Filip Van Immerseel, Richard Ducatelle, Francis Impens, Gunther Antonissen

**Affiliations:** 1 Department of Pathobiology, Pharmacology and Zoological Medicine, Faculty of Veterinary Medicine, Livestock Gut Health Team (LiGHT) Ghent, Ghent University, 9820 Merelbeke, Belgium; 2 VIB-UGent Center for Medical Biotechnology, VIB, 9052 Ghent, Belgium; 3 Department of Biomolecular Medicine, Ghent University, 9052 Ghent, Belgium; 4 VIB Proteomics Core, VIB, 9052 Ghent, Belgium; 5 Impextraco NV, Wiekevorstsesteenweg 38, 2220 Heist-op-den-Berg, Belgium

**Keywords:** Biomarkers, necrotic enteritis, gut health, broiler chicken, proteomics

## Abstract

Necrotic enteritis (NE) in broiler chickens is caused by the overgrowth of toxin-producing strains of *Clostridium (C.) perfringens*. This study aims to analyze the blood proteome of broiler chickens affected by NE, providing insights into the host’s response to the infection. Using MS/MS-based proteomics, blood plasma samples from broilers with necrotic lesions of different severity were analyzed and compared to healthy controls. A total of 412 proteins were identified, with 63 showing significant differences; for 25 of those correlation with disease severity was observed. Functional analysis revealed that proteins affected by NE were predominantly associated with the immune and signaling processes and extracellular matrix (ECM) structures. Notably, regulated proteins were significantly involved in bioprocesses related to complement activation, acute phase reaction, proteolysis and humoral immune response. The proteomics findings suggest that the changes in plasma proteins in response to NE are driven by the host’s intensified efforts to counteract the infection, demonstrating a.o. activation of ECM-degrading proteases (MMP2, TIMP2), acute phase response (HPS5, CP, EXFABP, TF, VNN) and notable reduction in basement membrane (BM) and ECM-related peptides (PLOD2, POSTN, COL1A1/2, HSPG2, NID2) detected in the blood of NE-affected birds. Moreover, the findings underscore a coordinated effort of the host to mitigate the *C. perfringens* infection via activating immune (a.o., C3, CFH, MASP2, MBL2) and acute phase (CP, ORM, TF, ExFAB) related proteins. This study provides a deeper understanding of the host–pathogen interactions and identifies potential biomarkers and targets for therapeutic intervention*.* Data are available via ProteomeXchange with identifier PXD054172.

**Supplementary Information:**

The online version contains supplementary material available at 10.1186/s13567-025-01519-7.

## Introduction

*Clostridium perfringens* is a gram-positive ubiquitous bacterium that is also part of the intestinal microbiota in chickens [1]. An intestinal overgrowth (i.e. enumeration ≥ 10^6^–10^8^ CFU/g) of toxin producing *C. perfringens* strains, however, can lead to compromised intestinal health, which, in extreme cases, may progress to necrotic enteritis (NE) [1]. NE is a complex multifactorial disease that primarily affects the small intestine. A combination of predisposing factors (e.g., coccidial infection*,* physical or environmental stress, feed changes, etc.) is required for the pathogen to elicit its full toxicogenic potential [2]. Animals affected by NE are usually characterized by altered integrity of the intestinal epithelial lining, imbalanced microbiome composition, impaired digestion and mucus secretion, increased feed transit time, all of which may ultimately result in mortality because of severe intestinal necrosis (clinical stage of NE). Numerous studies have addressed the pathogenesis of NE and its impact on host immunity, gastrointestinal morphology and microbiome landscape [1–4]. The NetB toxin plays a key role in NE development and the mode of action of the toxin is clear, but the target cells and cell receptors still remain to be determined [5]. In addition to the NetB toxin, a number of enzymes are playing a role in the pathogenesis, including mucinases that break down the mucus layer, and enzymes that affect the extracellular matrix, such as collagenases [6, 7].

To date, a few studies have investigated the plasma or serum proteome of chicken in response to infectious disease or inflammation [8, 9]. For instance, studies on immune responses and protein expression in e.g. liver, spleen, and cecum under different infection models—such as *Salmonella enterica* serovar Enteritidis infections in chickens- have been reported [9]. Horvatic et al. employed tandem mass tags (TMT) proteomic analysis of chicken plasma following lipopolysaccharide (LPS) administration to investigate endotoxin-induced inflammation, revealing key acute-phase proteins and biological pathways involved in the early innate immune response [8]. The effects of *Salmonella* Enteritidis challenge on the splenic proteomes of macrophages, heterophils [10] and T-lymphocytes [11] in chicken have also been detailed. While substantial advances in understanding the pathogenesis of *C. perfringens* associated NE and deciphering intestinal responses to the pathogen have been reported, there is limited research specifically examining blood proteome changes in response to NE [8, 12, 13].

Measuring blood-based proteins is a routine practice for detecting biomarkers of disease, enabling, for instance, early diagnostics, differentiation between infections with akin symptoms and tracking disease progression [14, 15]. Also, the blood proteome in response to infection can yield data that may help to understand the pathogenesis. As a biofluid “ubiquitous” within the body, blood plasma retains an amalgama of proteins released from various cells and tissues. Thus, changes in blood protein concentrations often reflect the response of the host to a particular pathological state and correlate with the degree of disease severity [14, 16]. While in human medical research numerous blood proteomics studies have focused on discovering biomarkers for diagnosis and prognosis, as well as for exploring the underlying mechanisms of diseases, limited research has been done in poultry [17, 18]. To our knowledge, the response of the chicken blood proteome to NE has not been studied so far. Currently, lesion scoring and mortality rates are commonly used to quantify NE severity. There is, however, a risk for misinterpretation, particularly with *Eimeria* predisposition models [19]. Addressing this knowledge gap can be useful to advance general understanding of host–pathogen interactions and identify biomarkers for the disease. The presented approach could not only identify potential biomarkers for NE but also aid in discovering protein markers linked to resilience, which could support selective breeding programs aiming at enhancing disease resistance. Ultimately, the identified potential biomarkers would require further validation in larger, diverse chicken populations to confirm their reliability and applicability in commercial settings.

## Materials and methods

**Figure 1:**
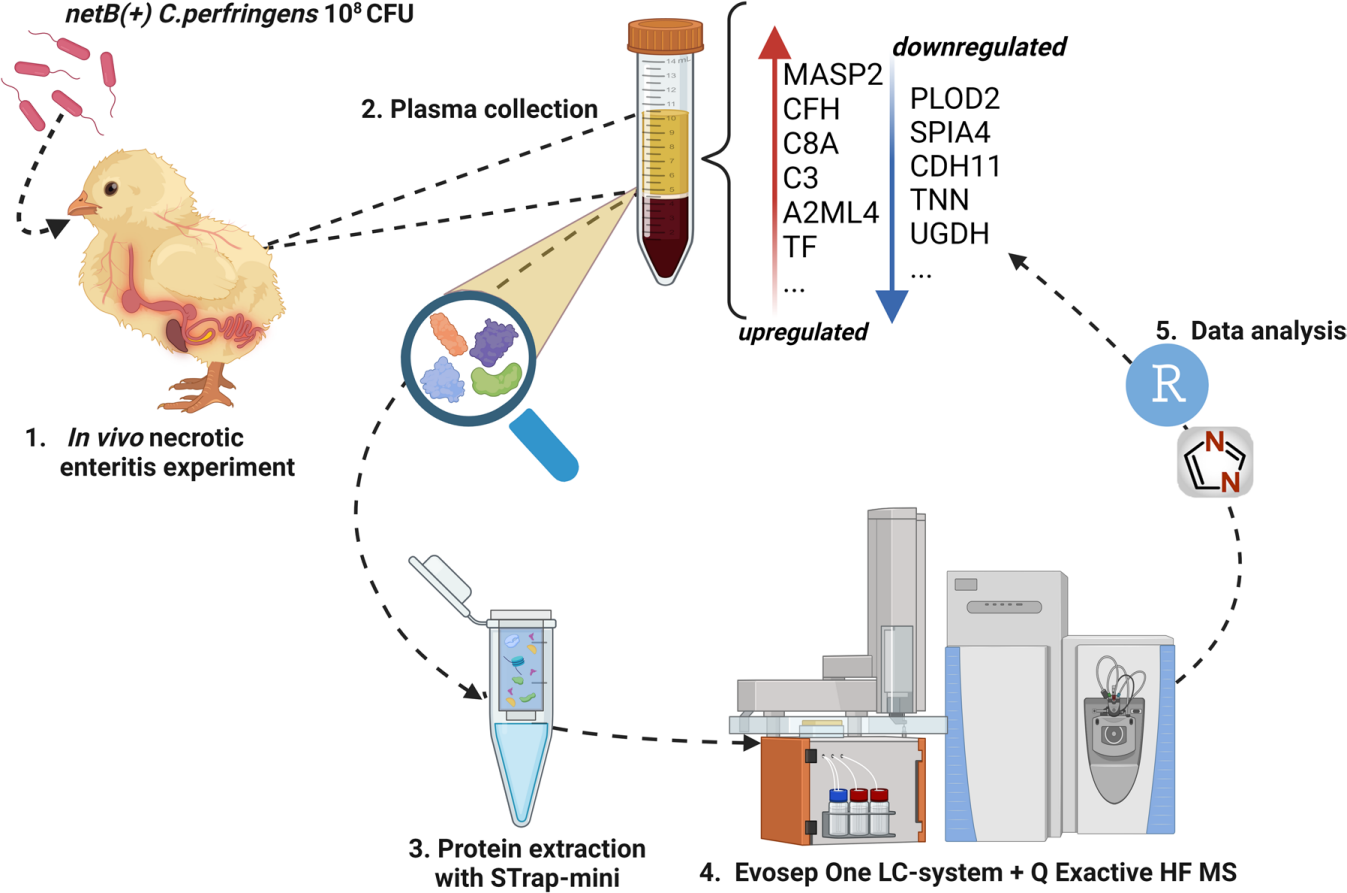
**Graphical abstract of the study.** This figure was created with Biorender.com and exported under a paid subscription.

All the experiments and manipulations with animals were approved by the ethical committee of the Faculties of Veterinary Medicine and Bioscience Engineering of Ghent University (EC2020-045). Housing and animal care was in accordance with the Royal Decree of 13 June 2010 following EU Directive 2007/43/EC of 28 June 2007. The experimental setup of the study is detailed in Figure 1.

### In vivo necrotic enteritis experiment

The in vivo model of necrotic enteritis (NE) was performed as previously described [20]. Briefly, a total of 192 one-day-old Ross 308 broiler chicks were randomly divided into two test groups with equal number of birds in each—challenge and control. All birds were housed in floor pens of 1 m^2^ each, allocating 12 animals per pen, 8 pens/test group, allowing free access to feed and water. All chickens received a wheat/rye-based diet (43%/7.5%) with soybean meal as a source of proteins, which was replaced with fishmeal (30%) from day 17 until the end of the experiment. This specific diet is rich in non-starch polysaccharides and proteins that serve as predisposing factors for the development of NE in chickens [20]. Chickens from the challenge group were administered a tenfold dose of live coccidiosis vaccine via oral gavage: Paracox-5 (containing 4 Eimeria species: *E. acervulina*, *E. maxima*, *E. mitis*, *E. tenella*) (MSD Animal Health, Brussels, Belgium), and Evant (containing 5 *Eimeria* species: *E. acervulina*, *E. maxima*, *E. mitis*, *E. praecox*, *E. tenella*) (Hipra, Girona, Spain) at days 14 and 16, respectively. During three consecutive days (day 18- day 20) animals were subsequently orally gavaged with approximately ${10}^{8}$ CFU of an overnight culture of pathogenic *netB-*positive *C. perfringens* type G strain (CP56) isolated from NE lesions. On day 21, prior to euthanizing, a sample of blood (3 mL) was withdrawn via venipuncture of the jugular vein from each chicken and collected into an EDTA-treated vacutainer followed by plasma separation (1900 rcf during 10 min at room temperature). Plasma samples were snap frozen in liquid nitrogen and kept at −70 °C.

At necropsy, the severity of NE was assessed by macroscopic evaluation of necrotic lesions along the small intestine (duodenum, jejunum and ileum), according to the scoring system described by Keyburn et al. [21]. Animals were categorized depending on the average developed lesion score: score 0 (no lesion), score 2 (mild), score 3–4 (sub-clinical NE), score 5–6 (clinical NE), and ctrl- (negative control, non-challenged birds). Chickens with a lesion score of 2 and above were considered NE positive.

For proteomic analysis, based on macroscopic lesion scoring results, a subset of 69 plasma samples that correspond to different NE lesion scores was selected:: “Ctrl”—samples of negative control group (non-challenged birds, *n* = 15), “lesion 0” representing animals that were challenged with *C. perfringens* but did not develop grossly visible NE lesions (*n* = 16), “lesion 2” as for samples with score 2 (*n* = 11), “lesion 3–4” representing score 3–4 (*n* = 15), and “lesion 5–6” corresponding to NE score 5–6 (*n* = 12). This sample size selection was estimated based on the findings of independent in vivo NE experiment performed previously and was guided by a multigroup analysis approach, allowing meaningful comparisons across lesion categories while prioritizing biological relevance and resource efficiency. The sample size was determined using the G*Power software (ver. 3.1.9.2, Kiel, Germany) with applied parameters of α = 0.05 and power = 95%.

### Plasma preparation for proteomics analysis

A comprehensive shotgun analysis of the plasma proteome was performed on samples from 69 broilers, categorized into five distinct groups based on lesion scores. Samples were prepared by suspension trapping, and subsequent LC–MS/MS analysis was conducted employing label-free DIA acquisition, as depicted in Figure 1.

One microlitre of each sample was added to 50 µL S-trap buffer containing 5% sodium dodecyl sulphate (SDS) and 50 mM triethylammonium bicarbonate (TEAB), pH 7.55. Following reduction by addition of 15 mM dithiothreitol and incubation for 30 min at 55 °C and alkylation by addition of 30 mM iodoacetamide and incubation for 15 min at RT in the dark, phosphoric acid was added to achieve a final concentration of 1.2%. Samples were diluted sevenfold with binding buffer containing 90% methanol in 100 mM TEAB, pH 7.1 and subsequently loaded on a 96-well S-Trap™ plate (ProtiFi, Fairport, NY, USA) by centrifugation at 1500 × *g* for 2 min at RT. Bound proteins were washed three times by addition of 200 µL binding buffer and centrifugation at 1500 × *g* for 2 min at RT. To initiate digestion of the proteins, 50 mM TEAB, pH 7.55 containing trypsin (1/100, w/w) was pipetted into each well. After overnight digestion at 37 °C, the S-Trap™ plate was centrifuged at 1500 × *g* for 2 min, and peptides underwent three-step elution in a deepwell receiver plate, first with 80 µL 50 mM TEAB, secondly with 80 µL 0.2% formic acid (FA) in water and finally with 80 µL 0.2% FA in water/acetonitrile (ACN) (50/50, v/v). The eluted peptides were dried completely by vacuum centrifugation and stored at −20 °C until further analysis.

### LC–MS/MS and data analysis

Dried peptides of all samples were redissolved in 20 µL loading solvent A, the peptide concentration was determined on a Lunatic spectrophotometer (Unchained Labs) and was adjusted to 0.015 µg/µL with 0.1% FA. iRT peptides (Biognosys, P/N Ki-3002-1) were added to all samples according to the instructions of the manufacturer. Then, 300 ng of each sample was loaded on Evotips (Evosep, P/N EV2003) according to the instructions of the manufacturer with a substitution of the wash step after sample loading by 2 wash steps with 80 µL 0.1% FA. All loaded Evotips were stored in 0.1% FA at 4 °C until LC–MS/MS analysis could be started. Samples were analysed on an Evosep One LC-system (Evosep, Odense, Denmark) in-line connected to a Q Exactive HF mass spectrometer (Thermo Fisher Scientific, Waltham, MA, USA). Peptides were separated with the 15 SPD method using the endurance Evosep column (15 cm × 150 µm I.D., 1.9 µm beads, Evosep). For elution of the peptides from the column 0.1% FA in LC–MS-grade water and 0.1% FA in ACN were used as mobile phases. The mass spectrometer was operated in data-independent mode (DIA), systematically isolating and fragmenting peptides in overlapping 10 m/z isolation windows. Full-scan MS spectra ranging from 375 to 1500 m/*z* with an AGC target value of 5 × 10^6^, a maximum fill time of 50 ms and a resolution at 200 m/z of 60 000 were followed by 30 quadrupole isolations with a precursor isolation width of 10 m/*z* for HCD fragmentation at an NCE of 30% after filling the trap at a target value of 3 × 10^6^ for maximum injection time of 45 ms. MS2 spectra were acquired at a resolution of 15 000 at 200 m/*z* in the Orbitrap analyser without multiplexing. The Skyline software tool was used to create the isolation intervals ranging from 400 to 900 m/*z* with an overlap of 5 m/z. Instrument longitudinal performance during the project was controlled using QCloud [22].

Raw files corresponding to the 69 DIA runs were searched together using the DIA-NN software (version 1.8.1) with mainly default settings [23]. Spectra were searched in library-free mode against the *Gallus gallus* canonical reference proteome sequences present in the UniProt database (database release version from June 2022), containing 18 112 sequences, with mainly default settings. A precursor mass range filter of 400–900 m/z was applied, match between runs (MBR) option was enabled and “mass accuracy” (MS2 mass accuracy) and “MS1 accuracy” (MS1 mass accuracy) parameters were set at 20 ppm and 10 ppm, respectively. A maximum of two trypsin missed cleavages and of five variable modifications (acetylation on protein N-termini or oxidation of methionine residues) was allowed. Peptide-to-protein assignment in DIA-NN follows a peptide-centric approach [23, 24] within which the software first identifies peptides by matching observed spectra to theoretical spectra generated from the UniProt database. It then applies a series of scoring and filtering steps, including the use of deep neural networks to distinguish between target and decoy peptides. Protein inference is performed by grouping peptides based on their protein associations. DIA-NN employs a conservative protein q-value calculation method to estimate the false discovery rate (FDR) at the protein level, considering only target and decoy precursors specific to each protein of interest. The software creates protein groups based on identified peptides, with special consideration given to proteotypic peptides. This process results in a filtered list of protein identifications, balancing sensitivity and accuracy in protein identification and quantification.

Further statistical analysis was performed using an in-house script in the R programming language (version 4.1.1) [25]. Protein expression matrices were prepared as follows: the DIA-NN main report output table was filtered at a precursor and protein library q-value cut-off of 1% and only proteins identified by at least one proteotypic peptide were retained. MaxLFQ-normalized protein intensities were log_2_-transformed and proteins with more than four valid values (at least 50% of samples in one experimental condition) in at least one experimental condition were kept, yielding a list of 443 quantified proteins. Imputation by random sampling from a normal distribution centered around the noise level (package DEP) was applied to assign missing values [26]. Multigroup comparison testing between the tested groups was performed using the limma package [27], with statistical significance for differential regulation set at an FDR cut-off value of 0.05.

Principal component analysis (PCA) was performed to further investigate the sample distances between the lesion score subtypes and control samples. For PCA, all quantified proteins for each chicken replicate were treated as one datapoint.

### Functional enrichment analysis

Functional analysis aiming to identify gene ontology (GO) terms associated with NE-induced proteomic changes was performed using Gene Set Enrichment Analysis (GSEA). Using the GSEA software (version 4.2.3) [28], gene names and associated F-statistic values were given as input for a GSEA pre-ranked analysis against all Gene Ontology (GO) annotation categories: biological process (BP), cellular component (CC) and molecular function (MF). An FDR* q*-value cut-off value of ≤ 0. 25 for enrichment significance was applied. A total of 329 out of the 412 quantified proteins were annotated and included in the analysis. The GO gene sets were obtained from GSEA database.

The protein–protein interaction (PPI) network for differentially abundant proteins was explored using the Search Tool for Retrieval of Interacting Genes database database (STRING, version 12.0, taxon identifier 9031) [29]. Clustering within analysed protein-coding gene set was based on “evidence” mode at high confidence, using MCL clustering at 1.8 inflation.

### Correlation analysis

The association between proteomics protein intensity and NE lesion score was evaluated using statistical software RStudio (version 4.1.1) [25] by performing Spearman rank correlation tests (cor.test function) and including a Benjamini–Hochberg correction for multiple testing.

## Results

### Necrotic enteritis induces changes in the blood proteome

**Figure 2:**
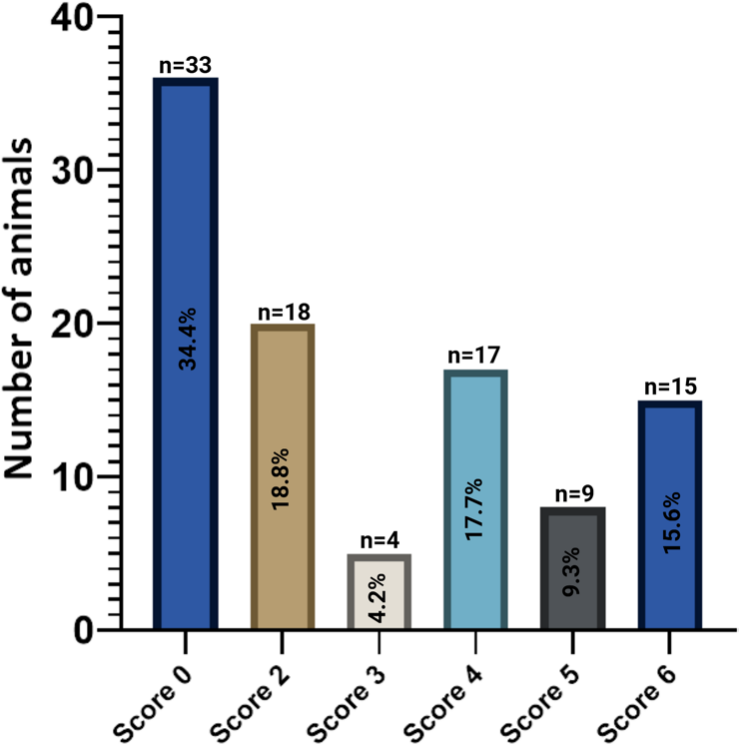
**Distribution of necrotic lesion scores among birds infected with C. perfringens during in vivo experiment.** The graph represents the number of birds that developed necrotic lesions post-infection, reflecting varying disease severity within the experimental flock: no necrotic lesions (score 0; *n* = 33); mild intestinal necrosis (score 2; *n* = 18); moderate necrotic enteritis (score 3 and 4; *n* = 4 and 17, respectively) or severe necrosis (score 5 and 6; *n* = 9 and 15, respectively). The total lesion score was calculated as average NE score across the middle part of duodenum, jejunum, ileum. Percentage values represent the proportion of the total number of birds challenged with *C. perfringens*. No significant effect due to allocation of the bird (pen effect) on the association between the treatment and the macroscopically observed lesion score was observed.

Changes in the chicken blood proteome resulting from necrotic enteritis were analysed using an necrotic enteritis (NE) model. During the in vivo experiment, three birds died before euthanasia and were excluded from the analysis. Macroscopic lesion scoring revealed that most of the challenged birds developed lesions (lesion score ≥ 2): a significant portion of the birds exhibited severe NE (lesion scores of 5 and 6, accounting for 29.5% and 24.4% of the birds, respectively), while a substantial number experienced mild NE (lesion scores of 3 and 4, representing 9% and 28.2% of the birds, respectively). The statistical analysis indicated lack of significant effect of animal localization in the experimental facility (pen effect) on the association between the type of treatment and the observed lesion score. Detailed results are provided in Figure 2.

**Figure 3:**
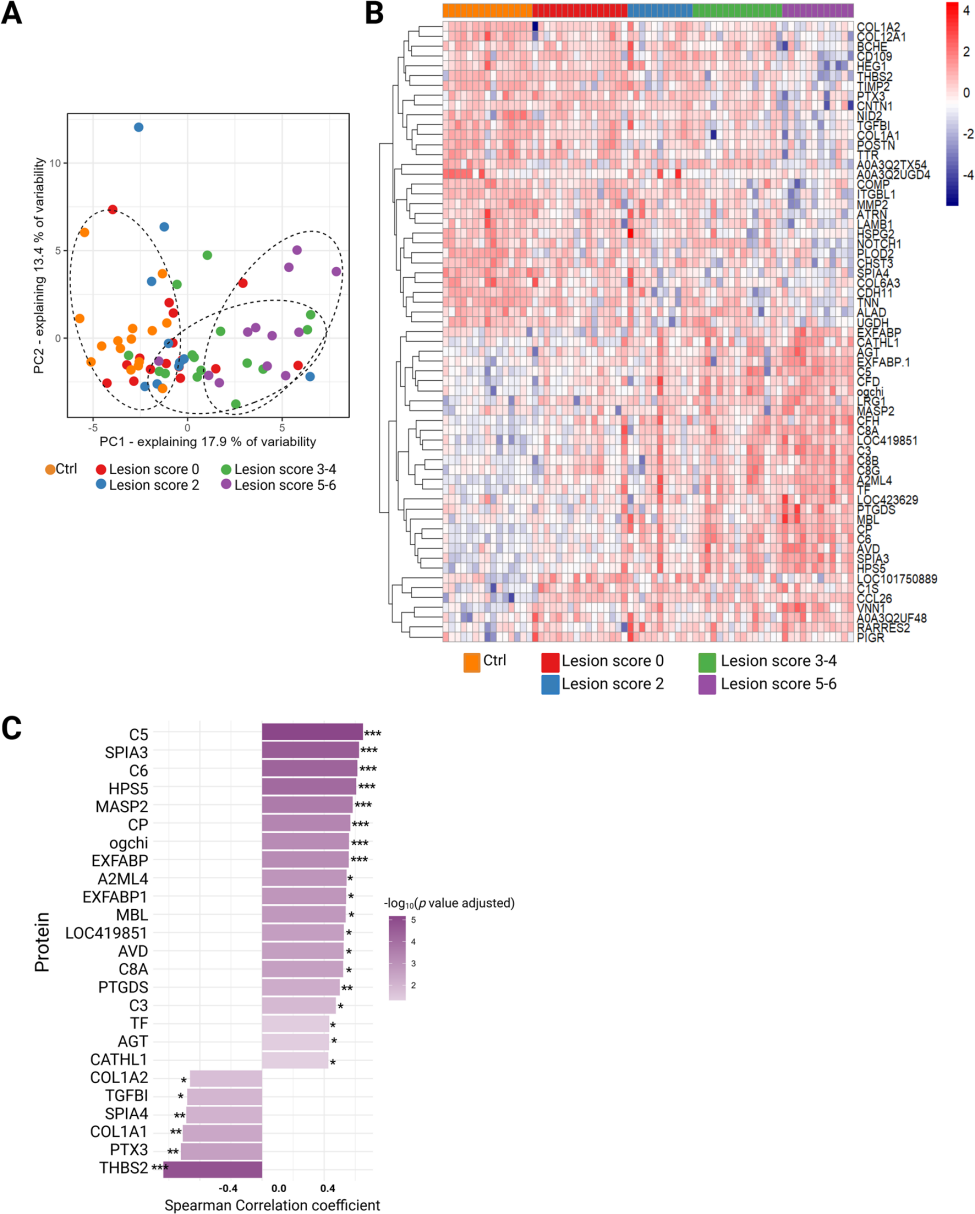
**Effects of necrotic enteritis infection in plasma proteome. A** Principal component analysis (PCA) of plasma samples based on proteomics. Plasma samples corresponding to assigned lesion score were mapped along the two principal components (PCs) and grouped together (dashed circles). For PCA, all quantified proteins identified per replicate were used as a variable; percentages of explained data variance for each PC are shown on the x and y axis. Each coloured circle represents a sample (*n* = 69).** B** Heatmap of z-scored expression values of differentially regulated proteins (limma F-test adj.*p*-value < 0.05) in each analysed sample, after hierarchical clustering. The colour key changes from red to dark blue to indicate the highest to the lowest protein expression. **C** Spearman correlation coefficients for the subset of differentially abundant proteins showing a statistically significant association between protein expression and NE severity (lesion score). Asterisks *,** and *** correspond to Spearman correlation test *adj.p-*values < 0.05, < 0.01, and < 0.001, respectively.

The MS-based proteomic methodology was applied in accordance with protocols validated in our laboratory (data unpublished). In summary, MS/MS analysis enabled detection of 6069 peptides that were further assigned to 443 proteins. PCA was performed on the replicate samples using all quantified proteins as variables clustered samples belonging to the same lesion score group together. The first principal component (PC1) explained 17.9% of the total variance, while the second principal component (PC2) explained 13.4% of the total variance (Figure 3A). The separation of chickens based on lesion scores is evident in the PCA plot, with chickens belonging to the same lesion score grouping together indicates the similarity of protein expression profiles depending on the NE severity, showing distinct clustering for the “lesion 5–6”-group compared to those with lower or no lesion.

### Different necrotic lesion scores lead to differentially regulated plasma proteins

Changes in the protein levels between lesion score group were assessed including all reliably quantified proteins (*n* = 412) (see Additional file 1). Ultimately, this enabled the discovery of 63 differentially abundant proteins, detected in plasma samples from minimum one biological condition (i.e. lesion score). The proteins of interest are listed in Figure 3B.

Among differentially abundant proteins, expression of ceruloplasmin (CP), serum amyloid protein (HPS5) and complement component 6 (C6) (F statistic > 15, see Additional file 2) were affected the most, highlighting larger variation of these proteins across “lesion score”-groups when compared to Ctrl. Serpin peptidase inhibitor 3 (SPIA3), alpha-2-macroglobulin-like 4 (A2ML4), complement component 4 binding protein (LOC419851) and chemokine (CCL26) showed the highest increase in response to the infection. In contrast, the plasma concentrations of, amongst others, contactin-N (CNTN1), heparan sulphate proteoglycan 2 (HPSG2) and carboxylic ester hydrolase (BCHE) were reduced in the challenged birds and differed notably across the lesion score groups (*adj. p-*value = 0.045, F statistic > 3, Additional file 2).

### Abundance of specific proteins correlates with the severity of necrotic enteritis

**Figure 4:**
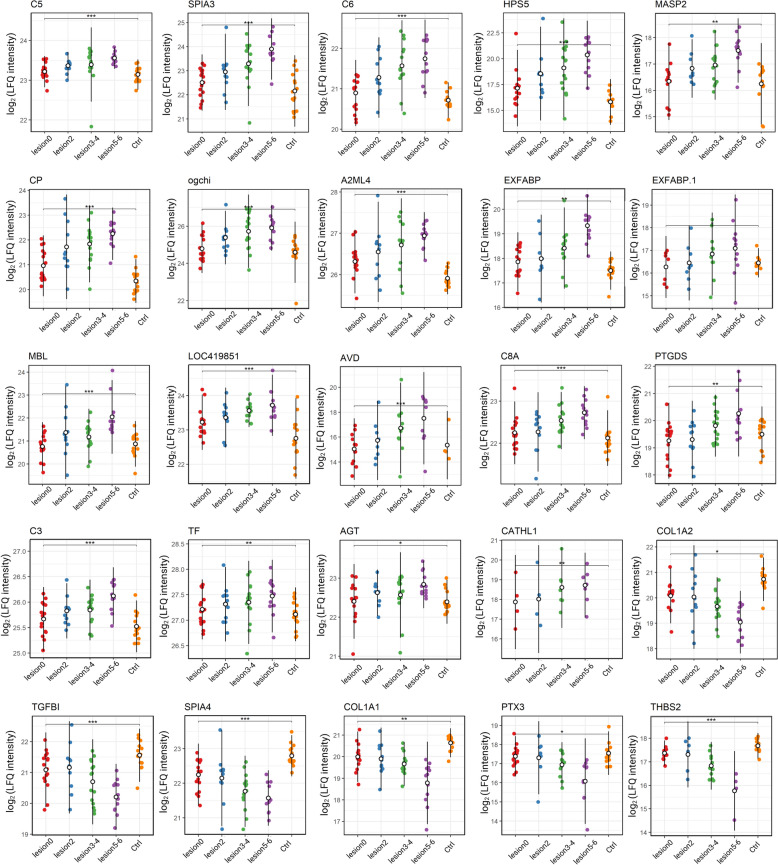
**Abundances of differentially regulated proteins that correlate with the disease severity.** Proteins were detected with MS-proteomics in blood plasma of healthy birds and birds challenged with necrotic enteritis. Each plot represents one of analysed proteins. Each dot corresponds to one animal per experimental condition. Asterisks *,** and *** represent statistical significance of *p* < 0.05, *p* < 0.01, and *p* < 0.001 between control and challenge group, respectively.

For the 63 differentially abundant proteins, correlation with the NE lesion score was evaluated using a Spearman rank correlation test (Figure 3C). A total of 25 proteins showed a significant correlation between their expression levels and the assigned NE lesion scores. Of these, six proteins (COL1A1, COL1A2, TGFBI, SPIA4, PTX3, THBS2) were negatively correlated with NE lesion scores (Spearman *rho* < -0.19, *adj. p-*value < 0.003), while 19 proteins (A2ML4, AGT, AVD, CATHL1, C3, C5, C6, C8A, CP, EXFABP, EXFABP1, HPS5, LOC419851, MASP2, MBL, ogchi, PTGDS, SPIA3, TF) exhibited a positive correlation with NE lesion scores (Spearman *rho* > 0.12, *adj. p-*value < 0.04). The results of correlation analysis are available in Additional file 2, intensity profiles of the correlating proteins across the NE severity groups are depicted on Figure 4.

### Functional categorization of differentially abundant plasma proteins

**Figure 5:**
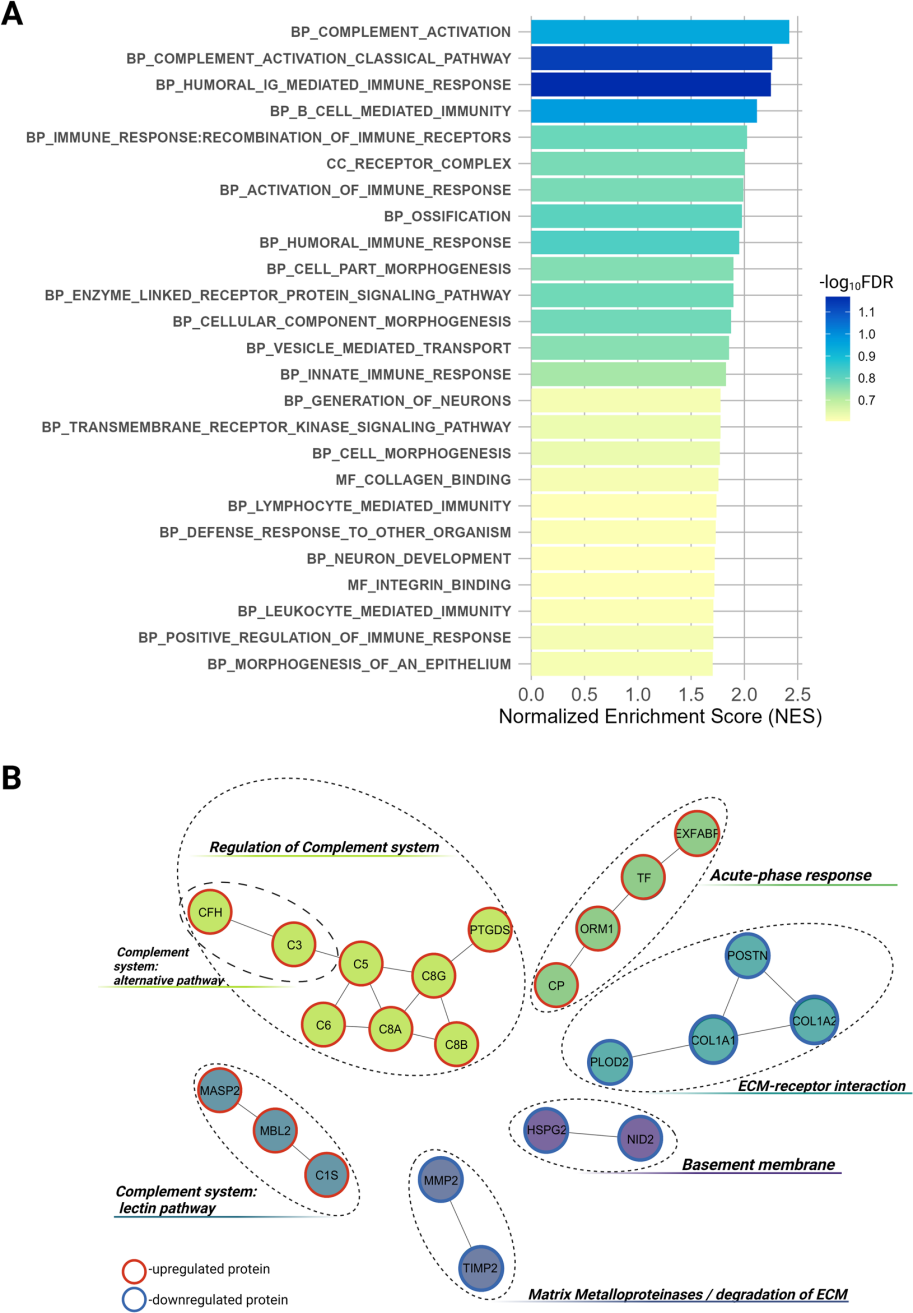
**Functional annotation analysis of differentially regulated proteins. A** Barplot of GSEA-based gene ontology (GO) enrichment analysis (FDR *q*-value cutoff of 0.25), detailing all terms associated with NE-induced chicken plasma proteome changes. Normalized enrichment score (NES) values are mentioned on x-axis, identified categories are mentioned on y-axis. The colours of the bars corresponds to the -log_10_ (FDR *q*-value). Classes of GO terms corresponding to BP: biological process, CC: cellular component, MF: molecular function are presented. **B** Network analysis (PPI) of the differentially expressed proteins**.** Six distinct functional clusters were detected in blood. Clustering within analysed protein-coding gene set was based on “evidence” mode at high confidence (> 0.9), using MCL clustering at 1.8 inflation. The red- and blue-framed symbols represent up- and down-regulation, respectively; each cluster is a set of highly-connected nodes and is illustrated in a discrete colour.

To explore the biological roles of proteins altered by necrotic enteritis, Gene Set Enrichment Analysis (GSEA) [28] was conducted using the statistics from differential abundance testing as metrics. The GSEA revealed 25 significantly enriched gene ontology terms significantly impacted by NE (positive enrichment score) (Figure 5A).

The majority of identified affected biological processes were related to immune responses, including humoral Ig-mediated immune response, complement activation (both classical and alternative), B cell-mediated immunity, and leukocyte-mediated immunity. The activation and regulation of both innate and adaptive immune responses were found to be significantly influenced by NE.

Additionally, considerable changes were identified in cell morphogenesis and development processes, including cellular component morphogenesis, cell part morphogenesis, and epithelial and neuron development. Impacted key pathways included enzyme-linked receptor protein signalling and transmembrane receptor kinase signalling pathways.

Molecular functions such as collagen and integrin binding were also significantly enriched, indicating changes in extracellular matrix (ECM) interactions. Furthermore, the cellular compartment most affected by NE was the receptor complex. Other notable bioprocesses affected by NE included ossification and vesicle-mediated transport. Detailed information regarding significantly enriched entries can be found in Additional file 3 online.

Finally, to provide a deeper understanding of synergy and functions of regulated proteins in NE, the STRING database was consulted. The PPI analysis revealed that proteins upregulated in NE clustered in functional sub-networks that are part of the acute phase response and complement system, while downregulated proteins clustered in sub-networks related to ECM, basement membrane (BM) and matrix metalloproteinases (Figure 5B).

## Discussion

*C. perfringens* is an enteropathogen that is able to induce disease in humans and animals [30]. Considering the critical role of balanced intestinal function and health in the welfare and performance of broilers, understanding how necrotic enteritis and its resolution affect the animal’s proteome is crucial for elucidating the pathogenesis and the host response to infection, and developing novel strategies to control this economically important disease.

Proteomics analysis identified 63 proteins with significantly different plasma concentrations, which varied notably across blood proteomes depending on the severity of NE. GSEA analysis revealed the enrichment of processes primarily involved in humoral immune response, complement activation, and cell signalling, indicating that NE impacts not only immune responses but also structural cellular integrity and function. Cell morphogenesis processes and collagen/integrin binding functions were found among principally affected pathways in GSEA, suggesting a crucial role of discovered proteins in maintaining the structural integrity and contributing to ECM-related processes. Using MCL clustering of differentially expressed proteins, six operational modules at a systems level were identified, associated with the acute phase response (APR), immune pathway activation, and structural processes involving the ECM and basement membrane (BM) (Figure 5B). Several studies have reported changes in ECM-associated plasma peptides often linked to tissue integrity and cell disruption [31–33]. In conditions affecting intestinal structure, the ECM was shown to undergo substantial changes involving both the restructuring of the matrix architecture and the synthesis of new ECM components [34].

Present proteomics findings reflect similar changes reported earlier in the literature: reduced plasma concentrations of peptides from important components of the ECM and BM e.g. THBS2, TNN, set of collagens (COL1A1; COL6A3; COL1A2), COMP, LAMB1, HSPG2, and LOC101750889), matrix-degrading proteases and their inhibitors (MMP2, TIMP2, SPIA3, SPIA4) [34, 35]. Interestingly, the abundance of ECM-associated proteins was notably decreased in animals experiencing severe NE (scores of 5–6) (Figure 3C). This reduction may be triggered by pathogenic *Clostridium* bacteria, or result from the resolution of acute inflammation, indicating that NE profoundly impacts the host at the cellular level by disrupting cell-ECM interactions and key signalling pathways. The differential abundance of other ECM components such as angiotensinogen (AGT), cartilage oligomeric matrix protein (COMP) and transforming growth factor-β (TGFB1), as suggested by Polansky et al. [36], is most likely facilitating leukocyte migration to infection sites and induce damage into the vascular system. Consequently, increase of AGT levels in plasma environment might lead to vasoconstriction, restricted serum diffusion, and oedema in affected tissues. Correlating with a disease severity (*rho* < 0.43, *adj. p-*value = 0.044), AGT upregulation in NE-infected birds was consistent with the findings of the host blood and liver response to *Salmonella* Enteritidis challenge [36]. Additionally, TGFB1 abundance was found negatively correlating with NE severity (*rho* < −0.33, *adj. p-*value = 0.01). Defective TGFB1 signalling was reported in men under inflammatory conditions of IBD [37] and in chickens challenged with *Salmonella* Enteritidis [36], while restoration of TGFB1 signalling has shown therapeutic potential in experimental intestinal inflammation models in mice [38].

Noteworthy, Martin and Smyth discovered that avian pathogenic *C. perfringens* strains are able to adhere to ECM molecules [30], particularly collagen, following structural damage due to preceding infiltration of intestinal epithelium by *Eimeria* parasites [39, 40], which results in exposure of ECM serving as binding sites for the pathogen, ultimately facilitating colonization [41–43]. Collagen-binding properties of *C. perfringens* are considered essential for further toxin secretion and NE development [30, 44, 45].

The BM, a specialized ECM produced by epithelial cells [46], supervises cell differentiation and organization, while ECM elements are primarily generated by mesenchymal cells (primarily myofibroblasts and fibroblasts) [47]. Therefore, reduced plasma levels of ECM and BM peptides may suggest altered fibroblast function during NE. A study on chicken fibroblasts demonstrated that these cells modulate the expression of immune-related genes in response to *Salmonella* Enteritidis [35, 48]. Additionally, (myo) fibroblasts have been considered a potential target of *C. perfringens* NetB toxin, as observed upon binding of recombinant NetB to subepithelial mesenchymal cells [49]. Although *netB* expression was not quantified in this study, it is important to note that used *C. perfringens* strain CP56 is a part of strain collection of the research group and has been previously confirmed to carry the *netB* plasmid in independent studies [50–53]. While this suggests the potential involvement of NetB-associated mechanisms, the specific contribution of the toxin to the observed proteomic responses in the blood of infected broilers remains to be fully elucidated. Heparan sulphate proteoglycan HSPG2 and nidogen NID2 which constitute BM and act as membrane-stabilizing agents were found at low abundance in the serum of NE birds, particularly with severe lesions [46]. This aligns with findings on NE development commencing at the basement membrane of epithelium [21, 54, 55], thereby initiating lesions [7].

Matrix metalloproteinases (MMPs) are expressed by inflammation-associated fibroblasts and are ECM-modulating enzymes [34]. In broilers, however, Van Damme et al., investigating the role of MMPs during necrotic enteritis, noted a reduction in the activity of these enzymes in the intestinal tissues of infected birds [56]. In contrast to the findings of Olkowski et al. [54] but consistent with the findings of Van Damme et al. [56], we found reduced plasma concentrations of both MMP-2 and tissue metalloproteinase inhibitor (TIMP-2), with particularly low levels in animals with severe NE. MMP-2 is a metalloproteinase (gelatinase) capable of destruction of type IV collagen, fibronectin and laminin—the main structural components of membrane synthesized by fibroblasts [57].

Notably, the observed lower abundance of ECM proteins may be attributed to selective proteolytic activity of endogenous circulating proteases, which preferentially induce changes in protein structure generating new protein N-termini (neo-N-termini) [58]. Under inflammatory conditions proteolysis can be exacerbated contributing to ECM degradation, potentially affecting its stability and biological function. As a result, the selective cleavage of ECM proteins may result in their fragments being underrepresented in MS analysis due to the limitations of tryptic digestion and database-dependent peptide identification [59]. This could explain the observed downregulation.

Abundance of periostin (POSTN)—a matricellular protein of fibroblast origin, implicated in supporting binding to the receptors on the cell surface and ECM adhesion [32, 60]- was found decreased in chickens with lesion score 2 and 5–6. In our findings, together with procollagen-lysine 5-dioxygenase (PLOD2) and collagens (COL1A1, COL1A2), POSTN composes a separate “ECM-receptor interaction” cluster (Figure 5B). Horvatić et al. demonstrated significantly fluctuating abundance of POSTN in broilers challenged with *Escherichia coli* lipopolysaccharide (LPS) [8]. Specifically, an initial small increase in POSN level, followed by decrease and finally an increase over a course of 12–72 h post-infection treatment, underscoring its dynamic response to LPS-induced challenge and potentially healing processes. Increased plasma levels of POSTN during remission in paediatric IBD was associated with its role in inflammation and mucosal repair [61]. The concentration of periostin in human serum is, however, age-dependent [61].

Proteins involved in the APR formed one of the key interacting modules in our PPI analysis (Figure 5B). The APR is a generalized immune response to local or systemic disruptions, infection, or inflammation, aimed at restoring homeostasis [62–64]. Several acute phase proteins (APPs), including alpha-1-acid glycoprotein (AGP/ ogchi), serum amyloid A (HPS5), avidin (AVD), fibronectin, ovotransferrin (TF) and ceruloplasmin (CP) [52, 62, 65, 66], were identified in our study and found to correlate with severity of NE. Identified AVD, EXFAB and HPS5, together with CATHL1, SPIA3, SPIA4, can be attributed to the activation of chicken macrophages and heterophils as reported by Sekelova et al. [10]. Comprising a part of innate immune reaction, positive stimulation of chicken several plasma proteins such as HPS5, AGP, TF, together with complement factor D (CFD) during an APR to LPS challenge was reported, with the levels peaking 12 h post-challenge [8]. Except for CFD, HPS5 (*rho* = 0.6, *adj. p-*value < 0.0001), oghi (AGP, *rho* = 0.55, *adj. p-*value = 0.001) and TF (*rho* = 0.43 *adj. p-*value < 0.001) abundances were found in agreement with increasing NE score. Moreover, AVD, HPS5, and EXFABP were identified as central markers in managing *Salmonella* Enteritidis infection in chickens both in spleen and cecum during systemic and localized infection, respectively [12]. Consequently, these proteins were suggested as potential of the immune response, with a possible role in reducing LPS levels, which may also decrease LPS concentration and associated inflammatory response. Nevertheless, as APPs are part of a non-specific stress response, their diagnostic value for intestinal diseases is limited as they show similar patterns of upregulation across various types of infections [16, 67].

Furthermore, the blood proteome in infected birds was marked by significant increases in regulatory proteins of the humoral response and in activators of the complement system (Figures 5A, B) [68]. Notably, elevated levels of proteins from all three pathways: classical pathway factors (C3, C5, C6, C8A, C8B, CFH, PTGDS), alternative pathway components (C3, CFH), and lectin pathway elements (mannose-binding lectin (MBL), C1, and MASP-2) were observed indicating a robust innate immune response in birds with NE. Complement activation through the MBL–MASP-2 lectin pathway complexes, identified as a separate interacting node (Figure 5B), aids in the opsonization and elimination of microorganisms, while C3-8 components help form membrane attack complexes (MAC) that disrupt pathogen cell walls [69]. In humans, the activation of the complement cascade has been recognized as a potential biomarker of IBD [70].

Finally, we identified a set of differentially abundant individual proteins with potential relevance to NE that did not fall into any GO-category or PPI cluster. Vascular non-inflammatory molecule-1 (VNN1), which was earlier introduced as positive APP, was related to decreased fatty acid metabolism in chickens infected with *Salmonella* Enteritidis [36]. Elevated levels of VNN1 were described to enhance cytoprotection during inflammation, which is possibly related to supply of cysteamine to tissue under conditions of oxidative stress [71, 72]. Taking into account the ability of *C. perfringens* to induce oxidative stress in the intestine [73], this could explain observed upregulation of VNN1 in the NE group in our study.

The polymeric immunoglobulin receptor (pIgR) is a glycoprotein specific to mucosal tissues that is secreted in crypts of the intestinal tract and, together with IgA, constitutes a part of intestinal mucosal immunity. In juvenile chickens, elevated levels of pIgR were related to colonization of the intestinal tract by the microbiota [74]. As this protein is of intestinal origin, monitoring its levels in blood could serve as assessment tool of mucosal immunity [75].

Found elevated in chickens with NE, leucine-rich alpha-2 glycoprotein (LRG1) is a crucial upstream signalling protein in the TGF-β signalling pathway, influencing various pathological processes through its interaction with TGF-β molecule [76, 77]. Its pronounced expression in inflamed UC and IBD tissues in humans correlated significantly with disease activity, though the exact mechanisms are not fully understood [78].

In conclusion, present findings suggest that changes observed in the plasma proteins upon mild and/or severe necrosis are induced due to the intensified physiological demands of the host to combat the infection. The magnitude of the proteome response reflects the severity of the disease, accentuating the systemic reaction of the chicken in counteracting the pathogen. Investigation of protein metabolism in response to systemic effects to different enteropathogenic infections in chickens could offer valuable insights. Finally, performing validation in samples from other enteric diseases in chickens or under field conditions will allow elucidating the potential use of these proteins as blood biomarkers.

## Supplementary Information

**Additional file 1. List of proteins (***n*** = 412) identified and quantified with DIA, with corresponding differential abundance analysis in NE lesion score groups vs Ctrl group.** Columns from left to right contain protein and gene IDs, protein name, *p*-value, adjusted (adj.) *p-*values, results of F statistic, significance label, average expression per experimental group, log_2_ LFQ expression values.

**Additional file 2. List of differentially regulated statistically significant proteins (***n*** = 63) with corresponding averaged differential abundance per tested group.** Columns from left to right contain protein and gene IDs, protein name, results of F statistic test, average protein expression per test group, results of Spearman rank correlation test (rho-, *p*-value, and adj.*p*-values, Significance). Asterisks next to the expression value indicate significance level when compared between NE lesion score group vs Ctrl; *-upregulation, **-downregulation.

**Additional file 3. Gene Set Enrichment Analysis (GSEA) for quantified protein-coding genes.** Columns from left to right represent classes of gene ontology (GO) terms (CC: cellular component, BP: biological process, MF: molecular function), number of genes in the gene set after filtering (SIZE), Enrichment Score (ES), normalized enrichment score (NES), nominal *p*-value (NOM p-val), and false discovery rate (FDR q-val).

## Data Availability

The mass spectrometry proteomics data have been deposited to the ProteomeXchange Consortium via the PRIDE [79] partner repository with the dataset identifier PXD05417 (Username: reviewer_pxd054172@ebi.ac.uk and Password: tnY2ixqJsRj0).

## Abbreviations

NE: necrotic enteritis
GSEA: Gene Set Enrichment Analysis
ECM: extracellular matrix
BM: basement membrane
DDA: data dependent acquisition
DIA: data independent acquisition
DTT: dithiothreitol
FA: formic acid
ACN: acetonitrile
PPI: protein–protein interaction
HPLC-MS: high performance liquid chromatography–mass spectrometry
PCA: principal component analysis
CP: ceruloplasmin
HPS5: serum amyloid protein
C6: complement component 6
SPIA3: serpin peptidase inhibitor 3
A2ML4: alpha-2-macroglobulin-like 4
LOC419851: complement component 4 binding protein
CNTN1: contactin-N
HPSG2: heparan sulphate proteoglycan 2
BCHE: carboxylic ester hydrolase
COL1A1: collagen α-1(I) chain
COL1A2: collagen α-2(I) chain
COL6A3: collagen α-3(VI) chain
TGFBI: transforming growth factor-ẞ-induced protein Ig-h3
SPIA4, PTX3, THBS2: thrombospondin-2
AVD: avidin
C3: complement component 3
C6: sixth complement component 6,
C8A: complement C8 α-chain
EXFABP: extracellular fatty acid-binding protein
MASP2: mannan binding lectin serine peptidase 2
MBL: mannose-binding protein
AGP/ogchi: alpha-1-acid glycoprotein, PTGDS
BP: biological process
CC: cellular components
MF: molecular function
MCL: Markov cluster algorithm
APR: acute phase response
TNN: tenascin N
COMP: cartilage oligomeric matrix protein
LAMB1: laminin subunit beta-1
LPS: lipopolysaccharide
HSPG2: heparan sulphate proteoglycan
MMP: matrix metalloproteinases
TIMP: 2-tissue metalloproteinase inhibitor-2
NID2: nidogen
POSTN: periostin
PLOD2: procollagen-lysine 5-dioxygenase
CD: Crohn’s disease
UC: ulcerative colitis
IBD: inflammatory bowel disease
APP: acute phase protein
OVT/TF: ovotransferrin
MAC: membrane attack complexes
VNN1: vascular non-inflammatory molecule-1
pIgR: polymeric immunoglobulin receptor
LRG1: leucine: rich alpha-2 glycoprotein
LOC101750889: platelet glycoprotein Ib α-chain

## References

[1] Timbermont L, Haesebrouck F, Ducatelle R, Van Immerseel F (2011) Necrotic enteritis in broilers: an updated review on the pathogenesis. Avian Pathol 40:341–347. 10.1080/03079457.2011.590967 21812711 10.1080/03079457.2011.590967

[2] Moore RJ (2016) Necrotic enteritis predisposing factors in broiler chickens. Avian Pathol 45:275–281. 10.1080/03079457.2016.1150587 26926926 10.1080/03079457.2016.1150587

[3] Fathima S, Shanmugasundaram R, Adams D, Selvaraj RK (2022) Gastrointestinal microbiota and their manipulation for improved growth and performance in chickens. Foods 11:1401. 10.3390/foods11101401 35626971 10.3390/foods11101401 PMC9140538

[4] Timbermont L, De Smet L, Van Nieuwerburgh F, Parreira RV, Van Driessche G, Haesebrouck F, Ducatelle R, Prescott J, Deforce D, Devreese B, Van Immerseel F (2014) Perfrin, a novel bacteriocin associated with netB positive *Clostridium perfringens* strains from broilers with necrotic enteritis. Vet Res 45:40. 10.1186/1297-9716-45-40 24708344 10.1186/1297-9716-45-40 PMC3992141

[5] Van Immerseel F, Lyhs U, Pedersen K, Prescott JF (2016) Recent breakthroughs have unveiled the many knowledge gaps in Clostridium perfringens -associated necrotic enteritis in chickens: the first International Conference on Necrotic Enteritis in Poultry. Avian Pathol 45:269–270. 10.1080/03079457.2016.1166857 27003036 10.1080/03079457.2016.1166857

[6] Awad MM, Ellemor DM, Bryant AE, Matsushita O, Boyd RL, Stevens DL, Emmins J, Rood JI (2000) Construction and virulence testing of a collagenase mutant of *Clostridium perfringens*. Microb Pathog 28:107–117. 10.1006/mpat.1999.0328 10644496 10.1006/mpat.1999.0328

[7] Van Damme L, Cox N, Callens C, Dargatz M, Flügel M, Hark S, Thiemann F, Pelzer S, Haesebrouck F, Ducatelle R, Van Immerseel F, Goossens E (2021) Protein truncating variants of colA in *Clostridium perfringens* Type G strains. Front Cell Infect Microbiol 11:645248. 10.3389/fcimb.2021.645248 33996628 10.3389/fcimb.2021.645248 PMC8117337

[8] Horvatić A, Guillemin N, Kaab H, McKeeganb D, O’Reilly E, Bainb M, Kuleša J, Eckersall PD (2019) Quantitative proteomics using tandem mass tags in relation to the acute phase protein response in chicken challenged with *Escherichia coli* lipopolysaccharide endotoxin. J Proteomics 192:64–77. 10.1016/j.jprot.2018.08.009 30114510 10.1016/j.jprot.2018.08.009

[9] Matulova M, Varmuzova K, Sisak F, Havlickova H, Babak V, Stejskal K, Zdrahal Z, Rychlik I (2013) Chicken innate immune response to oral infection with *Salmonella enterica* serovar Enteritidis. Vet Res 44:37. 10.1186/1297-9716-44-37 23687968 10.1186/1297-9716-44-37 PMC3663788

[10] Sekelova Z, Stepanova H, Polansky O, Varmuzova K, Faldynova M, Fedr M, Rychlik I, Vlasatikova L (2017) Differential protein expression in chicken macrophages and heterophils in vivo following infection with *Salmonella* Enteritidis. Vet Res 48:35. 10.1186/s13567-017-0439-0 28623956 10.1186/s13567-017-0439-0 PMC5473982

[11] Sekelova Z, Polansky O, Stepanova H, Fedr R, Faldynova M, Rychlik I, Vlasatikova L (2017) Different roles of CD4, CD8 and γδ T-lymphocytes in naive and vaccinated chickens during *Salmonella* Enteritidis infection. Proteomics 17:1700073. 10.1002/pmic.201700073 10.1002/pmic.201700073 28621911

[12] Matulova M, Rajova J, Vlasatikova L, Volf J, Stepanova H, Havlickova H, Sisak F, Rychlik I (2012) Characterization of chicken spleen transcriptome after infection with *Salmonella enterica* serovar Enteritidis. PLoS ONE 7:e48101. 10.1371/journal.pone.0048101 23094107 10.1371/journal.pone.0048101 PMC3477135

[13] Coble DJ, Sandford EE, Ji T, Abernathy J, Fleming D, Zhou H, Lamont SJ (2013) Impacts of *Salmonella enteritidis* infection on liver transcriptome in broilers. Genesis 51:357–364. 10.1002/dvg.22351 23097340 10.1002/dvg.22351

[14] Dey KK, Wang H, Niu M, Bai B, Wang X, Li Y, Cho JH, Tan H, Mishra A, High AA, Chen PC, Wu Z, Beach TG, Peng J (2019) Deep undepleted human serum proteome profiling toward biomarker discovery for Alzheimer’s disease. Clin Proteomics 16:16. 10.1186/s12014-019-9237-1 31019427 10.1186/s12014-019-9237-1 PMC6472024

[15] Anderson NL, Anderson NG (2002) The human plasma proteome. Mol Cell Proteomics 1:845–867. 10.1074/mcp.R200007-MCP200 12488461 10.1074/mcp.r200007-mcp200

[16] Ray S, Patel SK, Kumar V, Damahe J, Srivastava S (2014) Differential expression of serum/plasma proteins in various infectious diseases: specific or nonspecific signatures. Proteomics Clin Appl 8:53–72. 10.1002/prca.201300074 24293340 10.1002/prca.201300074 PMC7168033

[17] Deutsch EW, Omenn GS, Sun Z, Maes M, Pernemalm M, Palaniappan KK, Letunica N, Vandenbrouck Y, Brun V, Tao S, Yu X, Geyer PE, Ignjatovic V, Moritz RL, Schwenk JM (2021) Advances and utility of the human plasma proteome. J Proteome Res 20:5241–5263. 10.1021/acs.jproteome.1c00657 34672606 10.1021/acs.jproteome.1c00657 PMC9469506

[18] Ducatelle R, Goossens E, De Meyer F, Eeckhaut V, Antonissen G, Haesebrouck F, Van Immerseel F (2018) Biomarkers for monitoring intestinal health in poultry: present status and future perspectives. Vet Res 49:43. 10.1186/s13567-018-0538-6 29739469 10.1186/s13567-018-0538-6 PMC5941335

[19] Moore RJ (2024) Necrotic enteritis and antibiotic-free production of broiler chickens: challenges in testing and using alternative products. Anim Nutr 16:288–298. 10.1016/j.aninu.2023.08.012 38371475 10.1016/j.aninu.2023.08.012 PMC10869589

[20] Gholamiandehkordi AR, Timbermont L, Lanckriet A, Van Den Broeck W, Pedersen K, Dewulf J, Pasmans F, Haesebrouck F, Ducatelle R, Van Immerseel F (2007) Quantification of gut lesions in a subclinical necrotic enteritis model. Avian Pathol 36:375–382. 10.1080/03079450701589118 17899461 10.1080/03079450701589118

[21] Keyburn AL, Sheedy SA, Ford ME, Williamson MM, Awad MM, Rood JI, Moore RJ (2006) Alpha-toxin of *Clostridium perfringens* is not an essential virulence factor in necrotic enteritis in chickens. Infect Immun 74:6496–6500. 10.1128/IAI.00806-06 16923791 10.1128/IAI.00806-06 PMC1695520

[22] Chiva C, Olivella R, Borràs E, Espadas G, Pastor O, Solé A, Sabidó E (2018) QCloud: a cloud-based quality control system for mass spectrometry-based proteomics laboratories. PLoS One 13:e0189209. 10.1371/journal.pone.0189209 29324744 10.1371/journal.pone.0189209 PMC5764250

[23] Demichev V, Messner CB, Vernardis SI, Lilley KS, Ralser M (2020) DIA-NN: neural networks and interference correction enable deep proteome coverage in high throughput. Nat Methods 17:41–44. 10.1038/s41592-019-0638-x 31768060 10.1038/s41592-019-0638-x PMC6949130

[24] Ting YS, Egertson JD, Payne SH, Kim S, MacLean B, Kall L, Aebersol R, Smith RD, Stafford Noble W, MacCoss MJ (2015) Peptide-centric proteome analysis: an alternative strategy for the analysis of tandem mass spectrometry data. Mol Cell Proteomics 14:2301–2307. 10.1074/mcp.O114.047035 26217018 10.1074/mcp.O114.047035 PMC4563716

[25] R: The R Project for Statistical Computing. https://www.r-project.org/index.html.

[26] Zhang X, Smits AH, Van Tilburg GB, Ovaa H, Huber W, Vermeulen M (2018) Proteome-wide identification of ubiquitin interactions using UbIA-MS. Nat Protoc 13:530–550. 10.1038/nprot.2017.147 29446774 10.1038/nprot.2017.147

[27] Ritchie ME, Phipson B, Wu D, Hu Y, Law CW, Shi W, Smyth GK (2015) *limma* powers differential expression analyses for RNA-sequencing and microarray studies. Nucleic Acids Res 43:e47. 10.1093/nar/gkv007 25605792 10.1093/nar/gkv007 PMC4402510

[28] Subramanian A, Tamayo P, Mootha VK, Mukherjeed S, Ebert BL, Gillette MA, Paulovich A, Pomeroy SL, Golub TR, Lander ES, Mesirov JP (2005) Gene set enrichment analysis: a knowledge-based approach for interpreting genome-wide expression profiles. Proc Natl Acad Sci 102:15545–15550. 10.1073/pnas.0506580102 16199517 10.1073/pnas.0506580102 PMC1239896

[29] Szklarczyk D, Kirsch R, Koutrouli M, Nastou K, Mehryary F, Hachilif R, Gable AL, Fang T, Doncheva NT, Pyysalo S, Bork P, Jensen LJ, Mering C (2023) The STRING database in 2023: protein–protein association networks and functional enrichment analyses for any sequenced genome of interest. Nucleic Acids Res 51:D638–D646. 10.1093/nar/gkac1000 36370105 10.1093/nar/gkac1000 PMC9825434

[30] Martin TG, Smyth JA (2010) The ability of disease and non-disease producing strains of *Clostridium perfringens* from chickens to adhere to extracellular matrix molecules and Caco-2 cells. Anaerobe 16:533–539. 10.1016/j.anaerobe.2010.07.003 20654724 10.1016/j.anaerobe.2010.07.003

[31] Hinz B (2016) The role of myofibroblasts in wound healing. Curr Res Transl Med 64:171–177. 10.1016/j.retram.2016.09.003 27939455 10.1016/j.retram.2016.09.003

[32] Kinchen J, Chen HH, Parikh K, Antanaviciute A, Jagielowicz M, Fawkner-Corbett D, Ashley N, Cubitt L, Mellado-Gomez E, Attar M, Sharma E, Wills Q, Bowden R, Richter FC, Ahern D, Puri KD, Henault J, Gervais F, Koohy H, Simmons A (2018) Structural remodeling of the human colonic mesenchyme in inflammatory bowel disease. Cell 175:372-386.e17. 10.1016/j.cell.2018.08.067 30270042 10.1016/j.cell.2018.08.067 PMC6176871

[33] Younesi FS, Miller AE, Barker TH, Rossi FMV, Hinz B (2024) Fibroblast and myofibroblast activation in normal tissue repair and fibrosis. Nat Rev Mol Cell Biol 25:617–638. 10.1038/s41580-024-00716-0 38589640 10.1038/s41580-024-00716-0

[34] Brügger MD, Basler K (2023) The diverse nature of intestinal fibroblasts in development, homeostasis, and disease. Trends Cell Biol 33:834–849. 10.1016/j.tcb.2023.03.007 37080817 10.1016/j.tcb.2023.03.007

[35] Wong ZY, Nee E, Coles M, Buckley CD (2023) Why does understanding the biology of fibroblasts in immunity really matter? PLoS Biol 21:e3001954. 10.1371/journal.pbio.3001954 36745597 10.1371/journal.pbio.3001954 PMC9901782

[36] Polansky O, Seidlerova Z, Faldynova M, Faldynova M, Sisak F, Rychlik I (2018) Protein expression in the liver and blood serum in chickens in response to *Salmonella* Enteritidis infection. Vet Immunol Immunopathol 205:10–16. 10.1016/j.vetimm.2018.10.006 30458997 10.1016/j.vetimm.2018.10.006

[37] Monteleone G, Kumberova A, Croft NM, McKenzie C, Steer HW, MacDonald TT (2001) Blocking Smad7 restores TGF-β1 signaling in chronic inflammatory bowel disease. J Clin Invest 108:601–609. 10.1172/JCI12821 11518734 10.1172/JCI12821 PMC209401

[38] Strober W, Fuss I, Kitani A (2001) Regulation of experimental mucosal inflammation. Acta Odontol Scand 59:244–247. 10.1080/00016350152509274 11570528 10.1080/00016350152509274

[39] Gao Y, Sun P, Hu D, Tang X, Zhang S, Shi F, Yan X, Yan W, Shi T, Wang S, Zou S, Yin G, Liu X, Dong H, Suo X (2024) Advancements in understanding chicken coccidiosis: from *Eimeria* biology to innovative control strategies. One Health Adv 2:6. 10.1186/s44280-024-00039-x

[40] Van Immerseel F, Rood JI, Moore RJ, Titball RW (2009) Rethinking our understanding of the pathogenesis of necrotic enteritis in chickens. Trends Microbiol 17:32–36. 10.1016/j.tim.2008.09.005 18977143 10.1016/j.tim.2008.09.005

[41] Uzal FA, Freedman JC, Shrestha A, Theoret JR, Garcia J, Awad MM, Adams V, Moore RJ, Rood JI, McClane BA (2014) Towards an understanding of the role of *Clostridium perfringens* toxins in human and animal disease. Future Microbiol 9:361–377. 10.2217/fmb.13.168 24762309 10.2217/fmb.13.168 PMC4155746

[42] Arora S, Gordon J, Hook M (2021) Collagen binding proteins of gram-positive pathogens. Front Microbiol 12:628798. 10.3389/fmicb.2021.628798 33613497 10.3389/fmicb.2021.628798 PMC7893114

[43] Wade B, Keyburn AL, Haring V, Ford M, Rood JI, Moore RJ (2016) The adherent abilities of *Clostridium perfringens* strains are critical for the pathogenesis of avian necrotic enteritis. Vet Microbiol 197:53–61. 10.1016/j.vetmic.2016.10.028 27938683 10.1016/j.vetmic.2016.10.028

[44] Goo D, Park I, Nam H, Lee Y, Sawall J, Smith AH, Rehberger TG, Li C, Lillehoj HS (2023) Collagen adhesin protein and necrotic enteritis B-like toxin as biomarkers for early diagnosis of necrotic enteritis in commercial broiler chickens. Poult Sci 102:102647. 10.1016/j.psj.2023.102647 37060834 10.1016/j.psj.2023.102647 PMC10139936

[45] Wade B, Keyburn AL, Seemann T, Rood JI, Moore RJ (2015) Binding of *Clostridium perfringens* to collagen correlates with the ability to cause necrotic enteritis in chickens. Vet Microbiol 180:299–303. 10.1016/j.vetmic.2015.09.019 26455806 10.1016/j.vetmic.2015.09.019

[46] Pompili S, Latella G, Gaudio E, Gaudio E, Sferra R, Vetuschi A (2021) The charming world of the extracellular matrix: a dynamic and protective network of the intestinal wall. Front Med 8:610189. 10.3389/fmed.2021.610189 10.3389/fmed.2021.610189 PMC8085262 33937276

[47] McAnulty RJ (2007) Fibroblasts and myofibroblasts: their source, function and role in disease. Int J Biochem Cell Biol 39:666–671. 10.1016/j.biocel.2006.11.005 17196874 10.1016/j.biocel.2006.11.005

[48] Szmolka A, Wiener Z, Matulova ME, Varmuzova K, Rychlik I (2015) Gene expression profiles of chicken embryo fibroblasts in response to Salmonella Enteritidis infection. PLoS One 10:e0127708. 10.1371/journal.pone.0127708 26046914 10.1371/journal.pone.0127708 PMC4457728

[49] Hustá M (2022) Novel methods for *Clostridium perfringens* isolation and NetB toxin quantification, the causative agent of necrotic enteritis in broiler chickens. Ghent University Faculty of Veterinary Medicine, Merelbeke

[50] Hustá M, Ducatelle R, Van Immerseel F, Goossens E (2021) A rapid and simple assay correlates in vitro NetB activity with *Clostridium perfringens* pathogenicity in chickens. Microorganisms 9:1708. 10.3390/microorganisms9081708 34442787 10.3390/microorganisms9081708 PMC8400579

[51] Hustá M, Tretiak S, Ducatelle R, Ducatelle R, Van Immerseel F, Goossens E (2023) *Clostridium perfringens* strains proliferate to high counts in the broiler small intestinal tract, in accordance with necrotic lesion severity, and sporulate in the distal intestine. Vet Microbiol 280:109705. 10.1016/j.vetmic.2023.109705 36822035 10.1016/j.vetmic.2023.109705

[52] Goossens E, Debyser G, Callens C, De Gussem M, Dedeurwaerder A, Devreese B, Haesebrouck F, Flügel M, Pelzer S, Thiemann F, Ducatelle R, Van Immerseel F (2018) Elevated faecal ovotransferrin concentrations are indicative for intestinal barrier failure in broiler chickens. Vet Res 49:51. 10.1186/s13567-018-0548-4 29925427 10.1186/s13567-018-0548-4 PMC6011339

[53] Dierick E, Hirvonen OP, Haesebrouck F, Ducatelle R, Van Immerseel F, Goossens E (2019) Rapid growth predisposes broilers to necrotic enteritis. Avian Pathol 48:416–422. 10.1080/03079457.2019.1614147 31043060 10.1080/03079457.2019.1614147

[54] Olkowski AA, Wojnarowicz C, Chirino-Trejo M, Laarveld B, Sawicki G (2008) Sub-clinical necrotic enteritis in broiler chickens: novel etiological consideration based on ultra-structural and molecular changes in the intestinal tissue. Res Vet Sci 85:543–553. 10.1016/j.rvsc.2008.02.007 18359497 10.1016/j.rvsc.2008.02.007

[55] Olkowski AA, Wojnarowicz C, Chirino-Trejo M, Drew MD (2006) Responses of broiler chickens orally challenged with *Clostridium perfringens* isolated from field cases of necrotic enteritis. Res Vet Sci 81:99–108. 10.1016/j.rvsc.2005.10.006 16337982 10.1016/j.rvsc.2005.10.006

[56] Van Damme L, Cox N, Callens C, Haesebrouck F, Dargatz M, Ducatelle R, Van Immerseel F, Goossens E (2020) C. perfringens challenge reduces matrix metalloproteinase activity in the jejunal mucosa of Eimeria-infected broiler chickens. Vet Res 51:100. 10.1186/s13567-020-00825-6 32771049 10.1186/s13567-020-00825-6 PMC7414673

[57] Senior RM, Griffin GL, Fliszar CJ, Shapiro SD, Goldberg GI, Welgus HG (1991) Human 92- and 72-kilodalton type IV collagenases are elastases. J Biol Chem 266:7870–7875. 10.1016/S0021-9258(20)89530-1 1850424

[58] Prudova A, Auf Dem Keller U, Butler GS, Overall CM (2010) Multiplex N-terminome analysis of MMP-2 and MMP-9 substrate degradomes by iTRAQ-TAILS quantitative proteomics. Mol Cell Proteomics 9:894–911. 10.1074/mcp.M000050-MCP201 20305284 10.1074/mcp.M000050-MCP201 PMC2871422

[59] Olsen JV, Mann M (2004) Improved peptide identification in proteomics by two consecutive stages of mass spectrometric fragmentation. Proc Natl Acad Sci 101:13417–13422. 10.1073/pnas.0405549101 15347803 10.1073/pnas.0405549101 PMC518757

[60] Gauthier V, Kyriazi M, Nefla M, Pucino V, Raza K, Buckley CD, Alsaleh G (2023) Fibroblast heterogeneity: Keystone of tissue homeostasis and pathology in inflammation and ageing. Front Immunol 14:1137659. 10.3389/fimmu.2023.1137659 36926329 10.3389/fimmu.2023.1137659 PMC10011104

[61] Izuhara K, Nunomura S, Nanri Y, Ono J, Takai M, Kawaguchi A (2019) Periostin: an emerging biomarker for allergic diseases. Allergy 74:2116–2128. 10.1111/all.13814 30964557 10.1111/all.13814

[62] O’Reilly EL, Eckersall PD (2014) Acute phase proteins: a review of their function, behaviour and measurement in chickens. Worlds Poult Sci J 70:27–44. 10.1017/S0043933914000038

[63] Gruys E, Toussaint MJM, Niewold TA, Koopmans SJ (2005) Acute phase reaction and acute phase proteins. J Zhejiang Univ Sci B 6:1045–1056. 10.1631/jzus.2005.B1045 16252337 10.1631/jzus.2005.B1045 PMC1390650

[64] Jain S, Gautam V, Naseem S (2011) Acute-phase proteins: as diagnostic tool. J Pharm Bioallied Sci 3:118. 10.4103/0975-7406.76489 21430962 10.4103/0975-7406.76489 PMC3053509

[65] Chen J, Tellez G, Richards JD, Escobar J (2015) Identification of potential biomarkers for gut barrier failure in broiler chickens. Front Vet Sci 2:14. 10.3389/fvets.2015.00014 26664943 10.3389/fvets.2015.00014 PMC4672187

[66] Lynagh GR, Collins RA, Kaiser P (2000) Development and use of monoclonal antibodies to chicken fibronectin to show that the chicken hepatocellular carcinoma cell line, LMH, constitutively expresses fibronectin. Res Vet Sci 68:147–152. 10.1053/rvsc.1999.0352 10756132 10.1053/rvsc.1999.0352

[67] Pang T, Leach ST, Katz T, Day AS, Ooi CY (2014) Fecal biomarkers of intestinal health and disease in children. Front Pediatr 2:6. 10.3389/fped.2014.00006 24479111 10.3389/fped.2014.00006 PMC3904282

[68] Sina C, Kemper C, Derer S (2018) The intestinal complement system in inflammatory bowel disease: Shaping intestinal barrier function. Semin Immunol 37:66–73. 10.1016/j.smim.2018.02.008 29486961 10.1016/j.smim.2018.02.008

[69] Turner MW (2003) The role of mannose-binding lectin in health and disease. Mol Immunol 40:423–429. 10.1016/S0161-5890(03)00155-X 14568388 10.1016/s0161-5890(03)00155-x

[70] Krausgruber T, Fortelny N, Fife-Gernedl V, Senekowitsch M, Schuster LC, Lercher A, Nemc A, Schmidl C, Rendeiro AF, Bergthaler A, Bock C (2020) Structural cells are key regulators of organ-specific immune responses. Nature 583:296–302. 10.1038/s41586-020-2424-4 32612232 10.1038/s41586-020-2424-4 PMC7610345

[71] Berruyer C, Pouyet L, Millet V, Martin M, LeGoffic A, Canonici A, Garcia S, Bagnis C, Naquet F, Galland F (2006) Vanin-1 licenses inflammatory mediator production by gut epithelial cells and controls colitis by antagonizing peroxisome proliferator-activated receptor γ activity. J Exp Med 203:2817–2827. 10.1084/jem.20061640 17145956 10.1084/jem.20061640 PMC2118186

[72] Miller I, de Almeida AM, Eckersall PD (2021) Across the great divide: proteomics becoming an essential tool for animal and veterinary sciences. J Proteomics 241:104225. 10.1016/j.jprot.2021.104225 33857699 10.1016/j.jprot.2021.104225

[73] Li P, Liu C, Niu J, Zhang Y, Li C, Zhang Z, Guo S, Ding B (2022) Effects of dietary supplementation with vitamin A on antioxidant and intestinal barrier function of broilers co-infected with coccidia and *Clostridium perfringens*. Animals 12:3431. 10.3390/ani12233431 36496951 10.3390/ani12233431 PMC9740507

[74] Lammers A, Wieland WH, Kruijt L, Jansma A, Straetemans T, Schots A, Den Hartog G, Parmentier HK (2010) Successive immunoglobulin and cytokine expression in the small intestine of juvenile chicken. Dev Comp Immunol 34:1254–1262. 10.1016/j.dci.2010.07.001 20621117 10.1016/j.dci.2010.07.001

[75] Wieland WH, Orzáez D, Lammers A, Parmentier HK, Verstegen MW, Schots A (2004) A functional polymeric immunoglobulin receptor in chicken (*Gallus gallus*) indicates ancient role of secretory IgA in mucosal immunity. Biochem J 380:669–676. 10.1042/bj20040200 14992684 10.1042/BJ20040200 PMC1224204

[76] Muk T, Jiang P-P, Stensballe A, Skovgaard K, Sangild PT, Ninh Nguyen D (2020) Prenatal endotoxin exposure induces fetal and neonatal renal inflammation via innate and Th1 immune activation in preterm pigs. Front Immunol 11:565484. 10.3389/fimmu.2020.565484 33193334 10.3389/fimmu.2020.565484 PMC7643587

[77] Zou Y, Xu Y, Chen X, Wu Y, Fu L, Lv Y (2022) Research progress on leucine-rich alpha-2 glycoprotein 1: a review. Front Pharmacol 12:809225. 10.3389/fphar.2021.809225 35095520 10.3389/fphar.2021.809225 PMC8797156

[78] Naka T, Fujimoto M (2018) LRG is a novel inflammatory marker clinically useful for the evaluation of disease activity in rheumatoid arthritis and inflammatory bowel disease. Immunol Med 41:62–67. 10.1080/13497413.2018.1481582 30938267 10.1080/13497413.2018.1481582

[79] Perez-Riverol Y, Bai J, Bandla C, Garcıa-Seisdedos D, Hewapathirana S, Kamatchinathan S, Kundu DJ, Prakash A, Frericks-Zipper A, Eisenacher M, Walzer M, Wang S, Brazma A, Vizcaıno JA (2022) The PRIDE database resources in 2022: a hub for mass spectrometry-based proteomics evidences. Nucleic Acids Res 50:D543–D552. 10.1093/nar/gkab1038 34723319 10.1093/nar/gkab1038 PMC8728295

